# Growth Arrest Specific 2 Is Up-Regulated in Chronic Myeloid Leukemia Cells and Required for Their Growth

**DOI:** 10.1371/journal.pone.0086195

**Published:** 2014-01-21

**Authors:** Haixia Zhou, Yue Ge, Lili Sun, Wenjuan Ma, Jie Wu, Xiuyan Zhang, Xiaohui Hu, Connie J. Eaves, Depei Wu, Yun Zhao

**Affiliations:** 1 The First Affiliated Hospital, Jiangsu Institute of Hematology, Key Laboratory of Thrombosis and Hemostasis of Ministry of Health, Soochow University, Suzhou, Jiangsu Province, P.R. China; 2 Cyrus Tang Hematology Center, Soochow University, Suzhou, Jiangsu Province, P.R. China; 3 Terry Fox Laboratory, British Columbia Cancer Agency, Vancouver, Canada; University Hospital Hamburg-Eppendorf, Germany

## Abstract

Although the generation of BCR-ABL is the molecular hallmark of chronic myeloid leukemia (CML), the comprehensive molecular mechanisms of the disease remain unclear yet. Growth arrest specific 2 (GAS2) regulates multiple cellular functions including cell cycle, apoptosis and calpain activities. In the present study, we found GAS2 was up-regulated in CML cells including CD34^+^ progenitor cells compared to their normal counterparts. We utilized RNAi and the expression of dominant negative form of GAS2 (GAS2DN) to target GAS2, which resulted in calpain activity enhancement and growth inhibition of both K562 and MEG-01 cells. Targeting GAS2 also sensitized K562 cells to Imatinib mesylate (IM). GAS2DN suppressed the tumorigenic ability of MEG-01 cells and impaired the tumour growth as well. Moreover, the CD34^+^ cells from CML patients and healthy donors were transduced with control and GAS2DN lentiviral vectors, and the CD34^+^ transduced (YFP^+^) progeny cells (CD34^+^YFP^+^) were plated for colony-forming cell (CFC) assay. The results showed that GAS2DN inhibited the CFC production of CML cells by 57±3% (n = 3), while affected those of normal hematopoietic cells by 31±1% (n = 2). Next, we found the inhibition of CML cells by GAS2DN was dependent on calpain activity but not the degradation of beta-catenin. Lastly, we generated microarray data to identify the differentially expressed genes upon GAS2DN and validated that the expression of *HNRPDL*, *PTK7* and *UCHL5* was suppressed by GAS2DN. These 3 genes were up-regulated in CML cells compared to normal control cells and the growth of K562 cells was inhibited upon HNRPDL silence. Taken together, we have demonstrated that GAS2 is up-regulated in CML cells and the inhibition of GAS2 impairs the growth of CML cells, which indicates GAS2 is a novel regulator of CML cells and a potential therapeutic target of this disease.

## Introduction

Chronic myeloid leukemia (CML) is characterized by the formation of Philadelphia chromosome and the generation of *BCR-ABL* fusion gene to encode the oncoprotein with deregulated tyrosine kinase activity. Targeting CML cells with tyrosine kinase inhibitor (TKI) against BCR-ABL can effectively treat the patients in chronic phase, however the single agent does not cure the disease yet [1], 2. Therefore it is still urgent to obtain comprehensive molecular insights of CML cells and identify novel therapeutic targets in current research of CML.

Growth arrest specific 2 (GAS2) was first identified by Schneider C. *et al*. [3], which has been reported to possess multiple functions including the regulation of cell morphology, cell cycle, apoptosis and protease activity [4]–[12]. GAS2 is inactivated in tumour cells with the increased H3K27 trimethylation and the overexpression of GAS2 in MCF7 cells promotes p53 dependent apoptosis induced by etoposide, which suggests its tumour suppressor function [9]. GAS2 also protects cells from malignant transformation by promoting p53 dependent cellular senescence via inhibiting the activity of calpain to stabilize p53 [10]. On the contrary, the dominant negative form of GAS2 (GAS2DN) has the ability to release the inhibitory effect of both GAS2 and Calpastatin on calpain [12], and it inhibits the *in vitro* growth of HCT116 cells (colorectal cancer) by activating calpain to degrade beta-catenin [13]. Recently Huang W. *et al*. have reported that both RNAi against GAS2 and GAS2DN suppress the growth of BCR-ABL^+^ U937 cells and BCR-ABL^+^ murine myeloid progenitor cells through the GAS2/calpain/beta-catenin axis [14]. Thus GAS2 has dual function in human cancers depending on what substrate calpain degrades.

As a matter of fact, the deregulation of GAS2 has been reported in CML. Janssen JJ. *et al.* first demonstrated that *GAS2* was up-regulated when the disease progressed from chronic phase (CP) at diagnosis to blast crisis (BC) [15]. Radich JP. *et al*. found that *GAS2* was one of the most differentially expressed transcripts when comparing CD34^+^ cells from patients in CP to those in BC [16]. Moreover, Diaz-Blanco E. *et al*. suggested the expression of *GAS2* was higher in CD34^+^ cells from CML patients in CP compared to that from normal bone marrow (NBM) using microarray. However, the report did not provide the validation data [17].

In the present study, we compared the expression of GAS2 in chronic phase CML patients to that in healthy donors; we also addressed whether and how the deregulated GAS2 contributed to the growth of CML cells. These data have revealed a novel function of GAS2 in CML cells, and suggested GAS2 is a novel therapeutic target of this disease.

## Materials and Methods

### Cells and Culture Media

K562, MEG-01 and SW620 cells were purchased from the cell bank of Chinese Academy (www.cellbank.org.cn), which were maintained with RPMI1640 plus 10% FBS. The primary CML or normal adult bone marrow samples were collected with informed consent forms in the Department of Hematology, the First Affiliated Hospital, Soochow University. The clinical characteristics of these patients were summarized in Table S1 in File S1. After gradient centrifuge with Lympholyte®-H cell separation media (Cedarlane Laboratories, Burlington, NC, USA), the nucleated cells were yielded and then purified with human CD34 EasySep™ kit (Stem Cell Technologies, Vancouver, BC, Canada) following the instruction of the manufacturer.

### Ethics Statement

The samples of patients and healthy donors were collected with written informed consent, and the Ethical Committee of Soochow University approved the study as well as contents of the written consent.

All animal work was approved by the Animal Experimental Committee of Soochow University and performed in accordance with the National Institutes of Health Guidelines for the Care and Use of Laboratory Animals.

### RNA Extraction and Q-RT-PCR

RNAprep Pure Micro kit (Tiangen, Beijing, China) was used to extract RNA. During the procedure DNaseI (Life Technologies, Grand Island, NY, USA) treatment was applied to minimize the contamination with genomic DNA according to the manufacturer’s protocol. RNA was reversely transcribed with SuperScriptIII (Life Technologies) to generate the first strand of cDNA, and Q-RT-PCR was performed using SYBR Green PCR MasterMix with 7500 real time PCR system (Applied Biosystems, Foster City, CA, USA). Gene specific primers for Q-RT-PCR analysis were designed with online software (www.universalprobelibrary.com) and the sequences of these primers were summarized in the Table S2 in File S1.

### Western Blot

Protein samples were prepared with the protein lysate buffer (Beyotime, Shanghai, China) supplemented with 100 mM PMSF, and then the protein samples with same amount (15 µg/lane) were separated with SDS-PAGE and transferred to the Immobilon™ PVDF membrane (Millipore, Billerica, MA, USA) using Bio-Rad gel system (Bio-Rad, Hercules, CA, USA). The cytosol and nucleus protein samples were prepared with Nuclear and Cytoplasmic Protein Extraction Kit (P0027, Beyotimes) following the instruction of manufacturer. The blot was performed following the instructions of the suppliers of various antibodies, including anti-GAS2 (ab109762, Abcam, Cambridge, MA, USA), anti-HNRPDL (ab83215, Abcam), anti-beta catenin (ab22656, Abcam), anti-Histone H3 (AH433-1, Beyotimes) and anti-Tublin (T6074, Sigma, St Louis, MO, USA). The blot was developed with chemiluminescence substrate (ECL) (GE Healthcare Life Sciences, Piscataway, NJ) automatically (Kodak Medical X-Ray Processor 102).

### FACS Analysis of Protein Expression

The cells were treated with Cell Permeablization Kit (AN DER GRUB Bio Research GmbH, Austria) and then stained with primary and secondary antibodies for FACS analysis. In brief, 3×10^5^ cells were washed with PBS twice and then fixed with reagent A at room temperature for 15 min; after PBS washing the cells were incubated with reagent B; the cells were incubated with primary antibody at 4°C overnight and then incubated with secondary antibody at room temperature for 1 h; the cells were analyzed with FACS (Calibur, Becton-Dickinson, Franklin Lakes, NJ, USA) after PBS washing.

### Immunofluorescence Assay

1×10^5^ cells were transferred to coated slides (Thermo Fisher, Waltham, MA, USA) with a cytocentrifuge (Cytospin 4, Thermo Fisher). After the cells were air-dried for 20 min at room temperature, they were fixed with 2% paraformaldehyde and permeabilized in 0.1% Triton X-100 for 5 min. The cells were blocked in Tris-buffered saline with 5% bovine serum albumin for 30 min, and then incubated with primary antibody (1/100) overnight at 4°C in humidifying container. The cells were washed 3 times with PBS and incubated with proper secondary antibody (Multisciences, Hangzhou, China; 1/800) for 1 h. After the cells were washed 3 times with PBS, they were stained with Hoechst 33342 (Sigma) and covered with Prolong Gold Antifade reagent (Life Technologies). The images were finally captured and analyzed with a confocal microscope (FV1000MPE-share, Olympus, Japan). The control staining was done with same procedure except that the primary antibody was replaced with isotype control antibody with same dilution,and no signal was captured, which confirmed the specific staining of target proteins (GAS2 and beta-catenin).

### Calpain Assays

Calpain activity assays were conducted with a commercially available kit (Biovision, Mountain View, CA, US) following the instruction of the supplier. In brief, the cells were washed 3 times with PBS before protein extraction; and then equal amount of protein lysate was mixed with fluorescence-labeled substrate for fluorometric detection with a microplate reader (SpectraMax M5, Molecular Devices, Sunnyvale, CA, USA).

### Construction of Lentiviral Vector, Production of Lentivirus and Viral Transduction

The full-length GAS2 was amplified with primers, forward: 5′-aa gga tcc gga taa ata atg tgc act gct ctg-3′ and reverse: 5′-tt gga tcc tca ctt aat ttc ctt ctt agc ctt at-3′; and the truncated GAS2 (GAS2DN) was amplified with the primers, forward: 5′-aa gga tcc gga taa ata atg tgc act gct ctg-3′ and reverse: 5′-tt gga tcc tca ttc cag ctt tat caa acc agg ag-3′. The underlined sequences were recognized with *Bam*HI. GAS2 and GAS2DN were sub-cloned into the *Bam*HI site of a lentiviral vector from the laboratory of Dr. Christopher Baum (Department of Experimental Hematology, Hannover Medical School, Hannover, Germany), in which the transgene was driven by spleen focus forming virus promoter (pSFFV). The lentivirus delivered shRNAs against *GAS2*, *CALPAIN2* and *HNRPDL* together with the negative control (shNC) were from GenePharma Co, Ltd (Shanghai, China).

The viral production was performed with a standard method. In brief, 10 µg transgene plasmid, 6.5 µg ΔR (8.74), 3.5 µg VSV-G and 2.5 µg Rev were co-transfected into 293T cells with calcium precipitate method. Virus containing media (VCM) was collected 48 and 72 hrs after transfection. The titration of VCM was assayed with MEG-01 cells, and in a typical preparation the titration was as high as 5×10^6^ IU/ml (IU, Infection Unit). The VCM was stored at −80°C. The virus were further concentrated over 200 fold by additional ultracentrifugation with Swi-32 rotor at 25,000 rpm for 1.5 hrs in an ultracentrifuge (Optima-100K, Beckman Coulter, Indianapolis, IN, USA).

1×10^5^ K562 or MEG-01 cells were mixed with 50 to 100 µl VCM overnight, and then the cells were washed with PBS 3 times. These cells were cultured for another 3 days before purification of fluorescent cells with a cell sorter (AriaIII, BD). The CD34^+^ cells were pre-stimulated with SFM (serum-free media) plus a cocktail of cytokines including Flt-3 L (Flt-3 Ligand, 100 ng/ml), SCF (Stem Cell Factor, 100 ng/ml), IL-6 (20 ng/ml), IL-3 (20 ng/ml) and G-CSF (20 ng/ml) for 2 days, later the cells were transduced with concentrated lentiviruses on a fibronectin-coated plate for more than 6 hrs. Later these cells were washed with PBS and continuously cultured for 3 days before their purification with a cell sorter (AriaIII, BD) to isolate the YFP^+^ cells or CD34^+^YFP^+^ cells.

### Proliferation Assay for the Cells Cultured in Liquid Media

2×10^4^ sorted cells were plated into 24-well plate (2×10^4^ cells/1 ml/well), and then the viable cells were counted 3 and 6 days later with Trypan Blue exclusion staining.

### Xenoengraftments with Nude Mice

6–8 week old nude mice were purchased from Shanghai Laboratory Animal Center, the Chinese Academy of Science (SLACCAS, Shanghai, P.R. China), and then raised in the animal facility of Soochow University. 1×10^7^ transduced cells were injected subcutaneously, and then the growth of the tumours was observed closely. The mice were monitored every 2 days, and no mice died within the period of observation. The growth of tumour did not affect the activity of these mice severely, and all the mice bearing tumour were sacrificed 21 days after the injection in a CO_2_ chamber. The tumours were analyzed with luminescence detector (IVIS Lumina II, Caliper Life Sciences, Hopkinton, MA, USA) and then dissected to be fixed for H & E staining.

### Assays of Transactivities of Beta-catenin

The TOPflash and FOPflash reporter constructs were products of Millipore. RL-TK control plasmid was from Promega (Madison, WI, USA). 3 µg TOPflash or FOPflash plus 0.3 µg RL-TK were mixed together and delivered with Nucleofector™ device (Lonza, Basel, Switzerland) following the instruction of the manufacturer, and then the reporter activities were measure with Dual-Luciferase Reporter Assay System (Promega) in Luminoskan Ascent reader (Thermo Scientific). The measurements of TOPflash and FOPflash were first normalized with the detection of Renilla fluorescence; and then the ratio of TOPflash versus FOPflash was used to represent the transcriptional activity of beta-catenin.

### Colony-forming Cell Assay

The FACS purified primary cells were plated into methylcellulose media (MethoCult™ H4230, Stem Cell Technologies) plus a cocktail of cytokines, including SCF (50 ng/ml), IL-3 (20 ng/ml), IL-6 (20 ng/ml), GM-CSF (20 ng/ml), G-CSF (20 ng/ml) and EPO (3 IU/ml); the colonies were classified and numerated 14–16 days later.

### Microarray Analysis

3 biological replicates of control and GAS2DN transduced MEG-01 cells were harvested for RNA extraction and equal amounts of RNA were pooled to perform the microarray experiments with Affymetrix chip (Human U133 plus2.0) in Shanghai Biotechnology Corporation according to Affymetrix technical manual. This dataset has been disposed to Gene Expression Omnibus (GEO), and the accession number is GSE49184.

## Results

### GAS2 is Up-regulated in CML Cells

**Figure 1:**
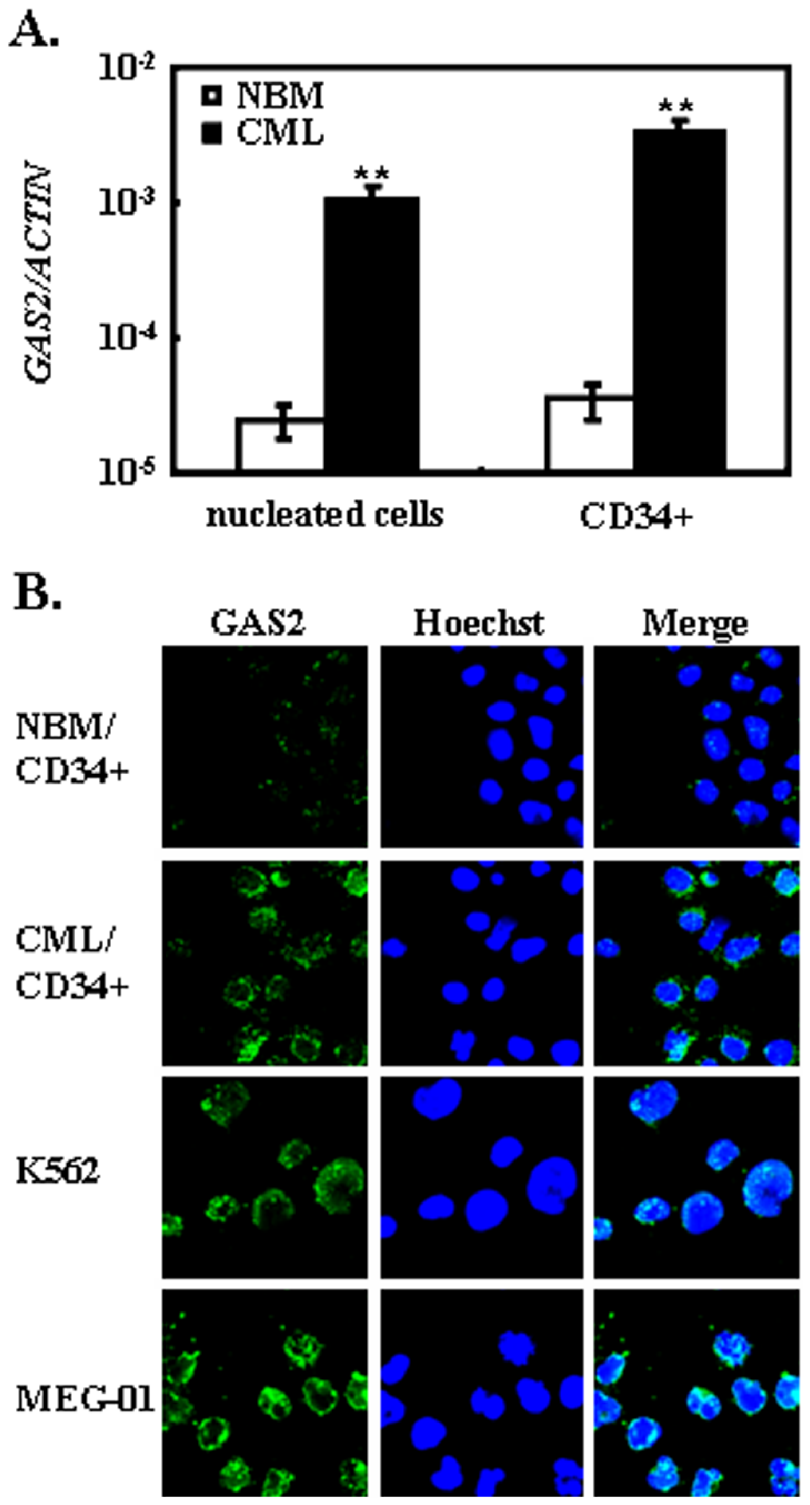
GAS2 is up-regulated in chronic myeloid leukemia. (A) The cells from chronic myeloid leukemia (CML) patients and healthy donors were collected and processed to yield nucleated cells, and then CD34^+^ cells were enriched with immunomagnetic method. The gene expression of *GAS2* was assessed with nucleated cells and CD34^+^ cells, respectively. For the nucleated cells, 7 healthy donors and 25 CML patients were recruited; for the CD34^+^ cells, 3 healthy donors and 8 CML patients were recruited. (B) Immunofluorescence assay was used to detect the expression of GAS2 (green) in CD34^+^ cells of normal bone marrow (NBM) and CML patient, together with K562 and MEG-01 cells. Hoechst 33342 was used to visualize the nuclei (blue). The representative graphs of individual section with confocal microscopy analyses were shown. Original magnification: ×150. The data were shown as mean ± S.E.M.; **mean *p*<0.01, which was estimated with student *t*-test in a two-tailed fashion.

Previous report has suggested the deregulation of *GAS2* in chronic phase CML; however the finding has not been validated yet [17]. In the present study, we collected nucleated cells from CML patients (n = 25) and healthy donors (n = 7) and CD34^+^ cells from CML patients (n = 8) and healthy donors (n = 3). The transcript expression of *GAS2* was significantly higher in CML patients compared to that in healthy donors. For nucleated cells, GAS2 was nearly 40-fold higher expressed in CML (*p*<0.01); and for CD34^+^ cells, GAS2 was about 90-fold higher expressed in CML as well (*p*<0.01, Figure 1A). Next, we analyzed the GAS2 protein expression with immuofluorescence method. The expression of GAS2 was hardly detected in CD34^+^ NBM cells, but it was readily detected in CD34^+^ CML cells, K562 and MEG-01 cells (Figure 1B). As expected, GAS2 resided in cytoplasm but not nuclei. The GAS2 protein was also up-regulated in nucleated cells from CML patients compared to those from healthy donors (data not shown).

### Targeting GAS2 Inhibits the Growth of CML Cells *in vitro* and *in vivo*

**Figure 2:**
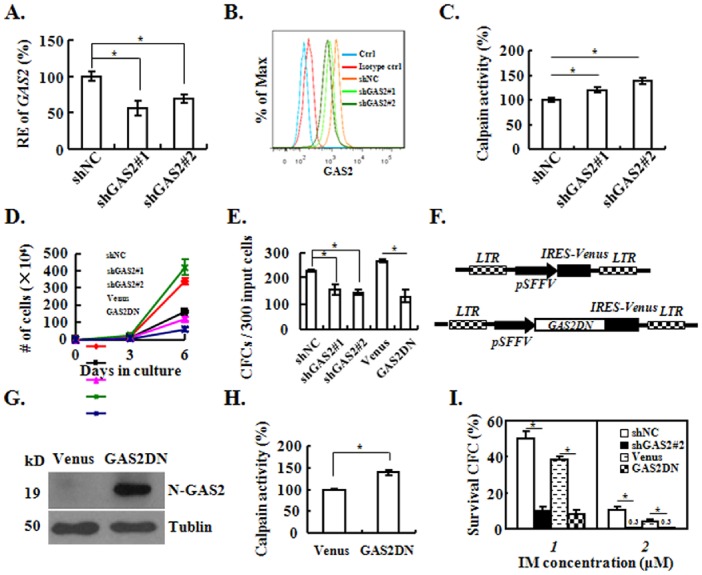
Targeting GAS2 with both RNAi and GAS2DN inhibits K562 cells. (A) The expression of *GAS2* was measured in shNC (control), shGAS2#1 and shGAS2#2 transduced cells with Q-RT-PCR. (B) The expression of GAS2 protein in shNC, shGAS2#1 and shGAS2#2 transduced cells were analyzed with FACS. (C) Calpain activities of shNC, shGAS2#1 and shGAS2#2 transduced cells were measured. (D) & (E) The proliferation and colony-forming cell (CFC) capacities of various virally transduced K562 cells were measured. (F) The schematic structure of lentiviral vector to express GAS2DN. LTR, long terminal repeat; pSFFV, spleen focus forming virus promoter; IRES, internal ribosome entry site; Venus, the enhanced yellow fluorescent protein; GAS2DN, the dominant negative form of GAS2. (G) The western blot was used to detect the expression of GAS2DN with an antibody recognizing N-terminus of GAS2 (N-GAS2). (H) Calpain activities of Venus and GAS2DN transduced cells were measured. (I) K562 cells were transduced with various lentiviral vectors. The FACS purified cells were plated in methylcellulose media with Imatinib mesylate (IM, final concentration as 1 µM and 2 µM), and then the colonies were numerated. The survival rates of CFC (IM treated versus IM untreated) of various transduced cells were calculated and compared. The data were shown as mean ± S.E.M. from at least 3 independent experiments; *mean *p*<0.05, which was estimated with student *t*-test in a two-tailed fashion.

**Figure 3:**
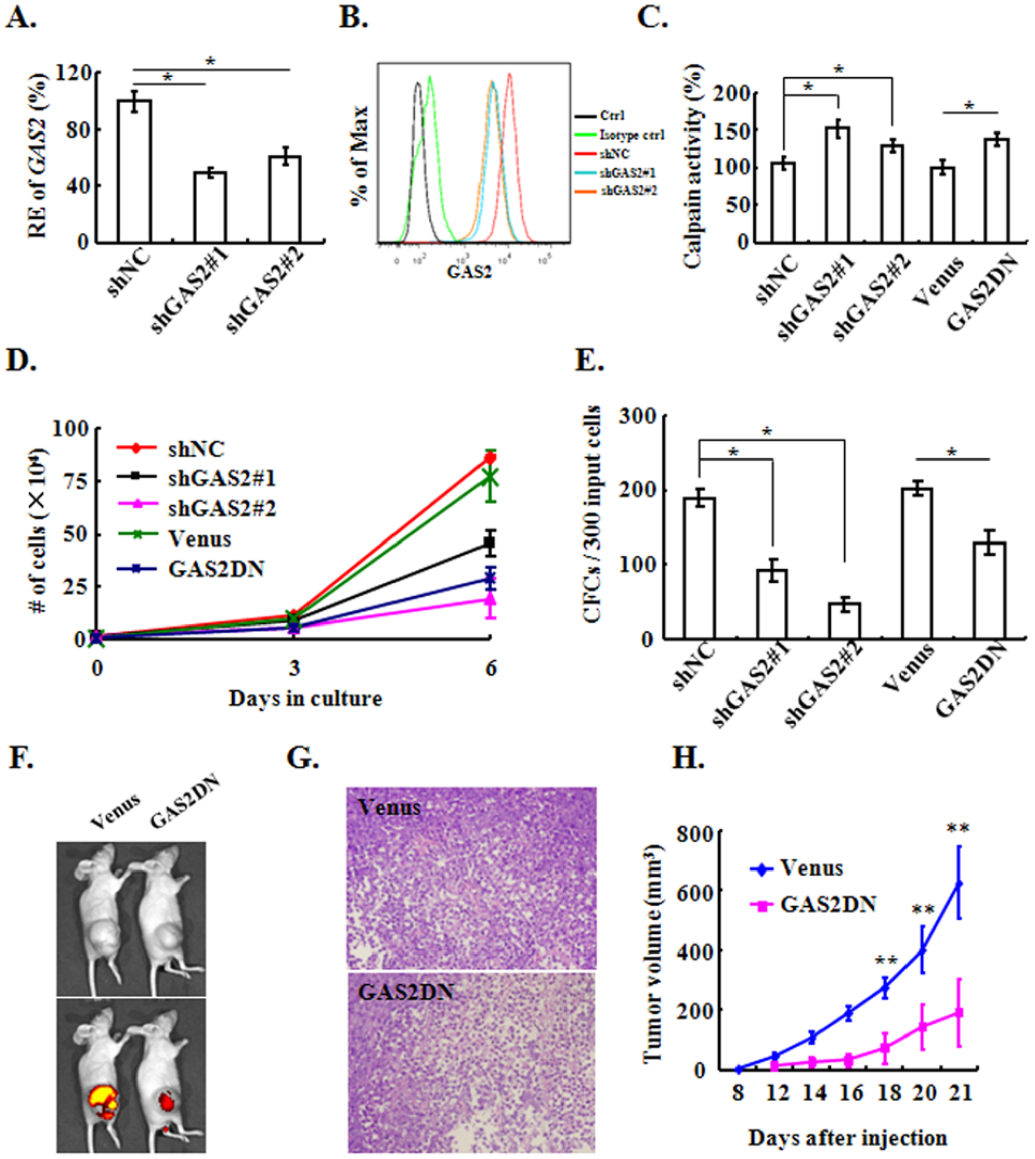
Targeting GAS2 suppresses the *in vitro* and *in vivo* growth of MEG-01 cells. (A) The expression of *GAS2* in shNC (control), shGAS2#1 and shGAS2#2 transduced MEG-01 cells was measured with Q-RT-PCR. (B) The expression of GAS2 protein in shNC, shGAS2#1 and shGAS2#2 transduced cells were analyzed with FACS. (C) The calpain activities of various virally transduced cells were measured. (D) & (E) The proliferation and CFC production of various virally transduced cells were assessed. (F) Tumour growth of the Venus and GAS2DN expressed cells in nude mice were observed, and the fluorescence was monitored with IVIS II imaging system as well. (G) Representative pictures of the H & E staining of the tumours were presented. (H) The average sizes of the tumours formed by the Venus and GAS2DN expressed cells were compared. The tumour size was estimated with the equation: volume = 1/2 × length × width^2^. Original magnification: ×200. *mean *p*<0.05 and **mean *p*<0.01, which were estimated with student *t*-test in a two-tailed fashion.

We firstly validated 2 independent sequences that suppressed the transcript expression of GAS2 by nearly 50% in both K562 and MEG-01 cells (Figure 2A, Figure 3A), and the GAS2 protein was suppressed by these two sequences as well (Figure 2B, Figure 3B). As expected, the calpain activity was elevated when GAS2 was knocked down (Figure 2C, Figure 3C). The silence of GAS2 resulted in the inhibition of proliferation in liquid culture and CFC production of both K562 and MEG-01 cells (Figure 2D & 2E, Figure 3D & 3E). Conversely, GAS2 overexpression was sufficient to rescue the suppressed CFC production induced by GAS2 silence in K562 cells (Figure S1 in File S1). GAS2Δ171–313 had the capacity to release the inhibitory effect of both GAS2 and Calpastatin against calpain, thus it was termed as the dominant-negative form of GAS2 (GAS2DN) [12]. As GAS2DN had the anti-cancer effect [13], [14], we constructed a lentiviral vector to express GAS2DN as well (Figure 2F). The expression of GAS2DN was validated with an antibody recognizing the N-terminus of GAS2 (N-GAS2), a band about 19 kD was detected as expected (Figure 2G). We also found that the calpain activities in GAS2DN expressed K562 and MEG-01 cells were elevated compared to those in the control cells (Figure 2H, Figure 3C). The proliferation of K562 and MEG-01 cells with the expression of GAS2DN was slower than the control cells; similarly the CFC production of K562 and MEG-01 cells with GAS2DN was less than that of the control cells (Figure 2D & 2E, Figure 3D & 3E). Both GAS2 silenced and GAS2DN expressed K562 cells with their control cells were exposed to IM, and targeting GAS2 sensitized the IM response of K562 cells (Figure 2I).

Given that both RNAi and GAS2DN inhibited the growth of leukemic cells, we started to perform experiments mainly with GAS2DN to study the efficacy and mechanism of the impaired CML cell growth induced by targeting GAS2.

In our hands, MEG-01 cells generate tumours more efficiently than K562 cells. Therefore 1×10^7^ control and GAS2DN expressed cells were injected subcutaneously into nude mice. 21 days later, tumours were observed in 10 out of 12 (83%) mice in control group; while tumours were found in only 3 out of 10 (30%) mice in GAS2DN group. All the tumours were visualized with IVIS imaging system to confirm that they were fluorescent, and they were all dissected and subjected to H & E staining, in which the leukemic cells were readily detected. The representative photos of fluorescent tumours and their H & E staining were presented (Figure 3F & 3G). In addition, GAS2DN not only reduced the frequency of tumour formation but also significantly reduced the growth rate of these tumours (Figure 3H).

**Figure 4:**
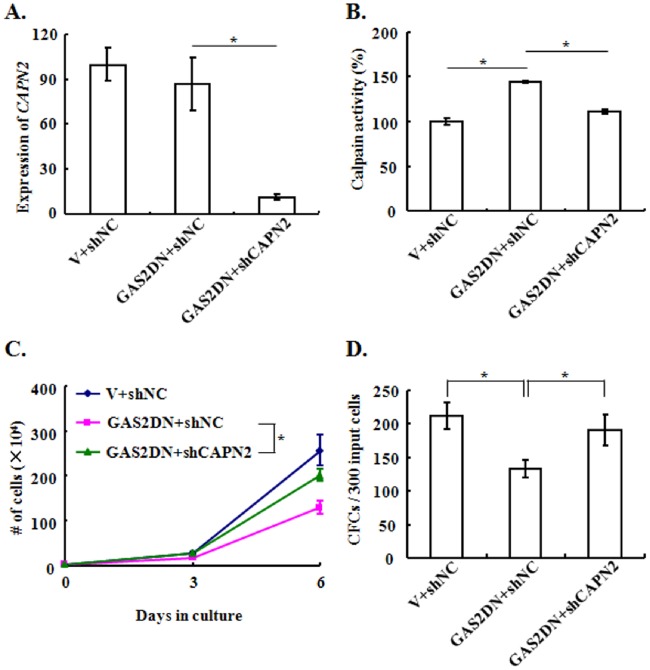
Silence of CALPAIN2 partically rescues the suppressed cell proliferation upon GAS2DN expression. In order to delineate the role of CALPAIN2 in the inhibitory effect of GAS2DN, a lentiviral vector to silence CALPAIN2 was used. (A) The mRNA expression of *CALPAIN2*, (B) activity of calpain, (C) cell proliferation in liquid culture and, (D) the CFC production of various transduced K562 cells were assessed. The data were shown as mean ± S.E.M. from more than 3 independent experiments; *mean *p*<0.05, which was estimated with student *t*-test.

To address whether the growth inhibition of K562 cells was calpain dependent, we utilized a lentiviral vector to silence CALPAIN2, which was capable to reduce the transcript expression of *CALPAIN2* by ∼ 90% and suppress the activity of calpain in both control and GAS2DN expressed K562 cells (Figure 4A & 4B, Figure S2 in File S1). The silence of CALPAIN2 rescued the inhibited proliferation in liquid culture and CFC production induced by GAS2DN (Figure 4C & 4D). The remaining calpain activity in GAS2DN plus CALPAIN2 silenced cells was roughly 10% higher than that of the control cells (p<0.05), which possibly explained why CALPAIN2 silence did not fully rescue the repressed proliferation caused by GAS2DN expression. In addition, we added PD150606 (calpain inhibitor) into the culture media and found the GAS2DN transduced cells with the inhibitor grew significantly faster than the untreated GAS2DN transduced cells. However this inhibitor affected the growth of control cells significantly, which suggested that the elevated cell growth of GAS2DN transduced cells in presence of PD150606 was underestimated (Figure S3 in File S1).

### GAS2DN Inhibits the CFC Production of Leukemic Progenitors

**Figure 5:**
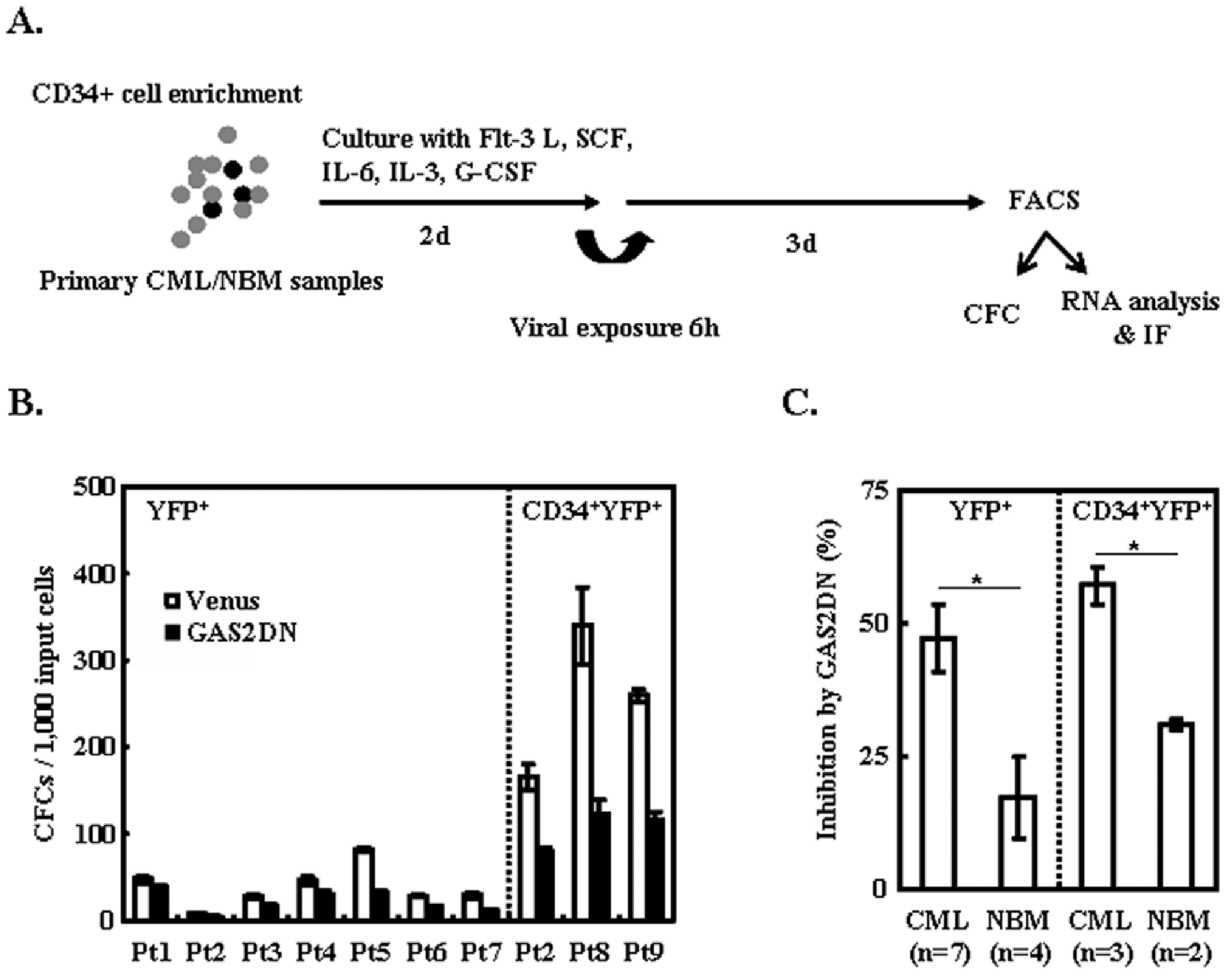
GAS2DN inhibits the CFC production of CML progenitor cells. (A) The CD34^+^ cells were enriched with immunomagnetic approach, and then were pre-stimulated with SFM (serum-free media) plus a cocktail of cytokines including Flt-3 L (Flt-3 Ligand), SCF (Stem Cell Factor), IL-6, IL-3 and G-CSF for 2 days, later the cells were transduced with lentiviral vectors on a fibronectin-coated plate for 6 hrs. After washing with PBS, these cells were cultured for additional 3 days and then purified with a cell sorter. (B) The total CFC yielded from Venus and GAS2DN transduced (YFP^+^) cells and CD34^+^ transduced (CD34^+^YFP^+^) cells were plotted. (C) The inhibition of CFC production of CML and NBM cells upon GAS2DN were compared. NBM, normal bone marrow. *mean *p*<0.05, which was estimated with student *t*-test in a two-tailed fashion.

To investigate the effect of GAS2DN on CD34^+^ cells from CML patients, GAS2DN was delivered with concentrated lentivirus, and then either YFP^+^ or CD34^+^YFP^+^ cells were purified with FACS and used for further cellular and molecular analysis (Figure 5A, Figure S4 in File S1). In summary, we found GAS2DN inhibited the CFC production of CML progenitor cells significantly (38±9 versus 21±5 colonies/1,000 input YFP^+^ cells, n = 7, *p* = 0.02; 250±50 versus 106±14 colonies/1,000 input CD34^+^YFP^+^ cells, n = 3, *p* = 0.05, Figure 5B). Moreover, we found that the CFC production inhibition induced by GAS2DN for CML cells was significantly higher than that for NBM cells (47±6% versus 17±8% for YFP^+^ cells, *p* = 0.02; 57±3% versus 31±1% for CD34^+^YFP^+^ cells, *p* = 0.01, Figure 5C), which demonstrated that GAS2DN had a stronger inhibitory effect on CFC production of CML progenitor cells than that on CFC production of normal progenitor cells.

### Targeting GAS2 does not Affect the Expression and Activity of Beta-catenin

**Figure 6:**
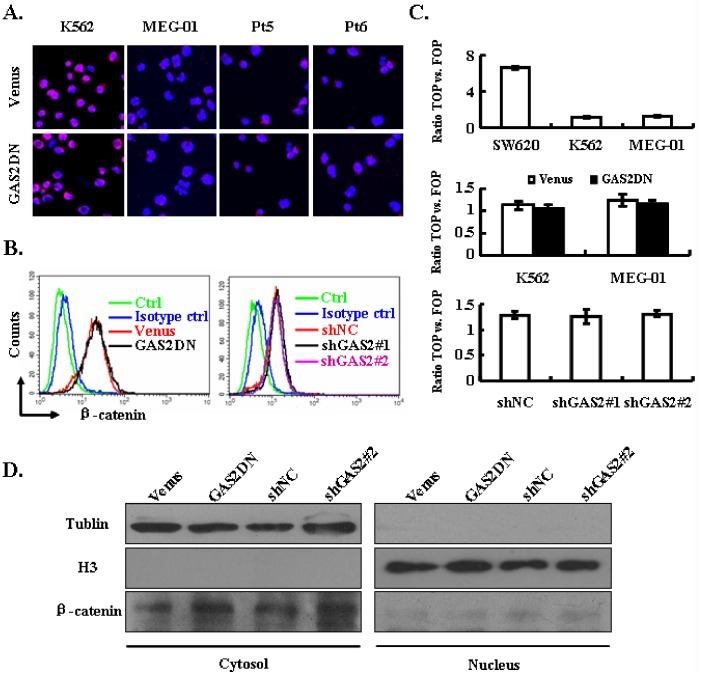
The expression and activity of beta-catenin are not affected by GAS2 targeting. (A) Immunofluorescence assay was used to detect the expression of beta-catenin (red) in Venus and GAS2DN transduced cells with Hoechst 33342 to visualize the nuclei (blue). The representative graphs of individual section with confocal microscopy analyses were shown. (B) The expression of beta-catenin was assessed quantitatively with flow cytometry in various virally transduced K562 cells. (C) The transcription activity of beta-catenin was measured in SW620 (human colorectal adenocarcinoma cell, as a positive control of beta-catenin activity), K562 and MEG-01 cells (upper panel), Venus and GAS2DN transduced K562 and MEG-01 cells (middle panel), and shNC, shGAS2#1 and shGAS2#2 transduced K562 cells (lower panel). The beta-catenin activated reporter vector (TOPflash) or its mutant vector (FOPflash) plus renilla reporter vector were used for transfection. The ratio of normalized TOPflash versus FOPflash was used to represent the activity of beta-catenin. (D) The cytosol and nucleus protein from various virally transduced cells were purified, and then subjected to immunoblot with antibodies against Tublin, beta-catenin and histone H3, respectively. The representative graph of 3 independent experiments was displayed.

The previous reports have demonstrated that calpain mediated beta-catenin degradation is key for the inhibitory effect of GAS2DN on cancer cells [13], [14]. To understand the molecular mechanism of how GAS2DN inhibited the growth of CML cells, we first detected the expression of beta-catenin in control and GAS2DN expressed cells including K562, MEG-01 and primary CML cells with immunofluorescence. We found beta-catenin was not equally expressed in these cells and the expression in primary CML cells was weak. Unexpectedly, the expression of beta-catenin was not altered upon GAS2DN in all tested cells (Figure 6A). Next, we measured the expression of beta-catenin quantitatively with FACS. Similarly, the expression of beta-catenin was not affected by GAS2DN expression or silence of GAS2 in K562 cells (Figure 6B). In addition, we purified cytosol and nucleus proteins with GAS2DN expressed and GAS2 silenced K562 cells with their control cells. The expression of beta-catenin was analyzed with immunoblot, and the expression of beta-catenin in both fractions was not changed upon GAS2DN expression or GAS2 silence either (Figure 6D). Lastly, we measured the transcription activity of beta-catenin in SW620, K562 and MEG-01 cells with TOPflash/FOPflash reporter plasmids. The transcriptional activity of beta-catenin was readily detected in SW620 cells, but not in K562 and MEG-01 cells (Figure 6C upper panel), and the activity was not affected by GAS2DN in both K562 and MEG-01 cells (Figure 6C middle panel). In addition, we were not able to detect the alteration of beta-catenin activity when GAS2 was knocked down either (Figure 6C lower panel). Collectively, these data suggested that inhibition of GAS2 might utilize other ways rather than beta-catenin degradation to suppress the growth of CML cells.

### Identification of the Differentially Expressed Transcripts upon GAS2DN

To address the molecular mechanism utilized by GAS2DN to inhibit CML cell growth with transcriptome approach, we collected 3 independent biological samples of MEG-01 control and GAS2DN expressed cells to extract RNA samples, and then the equal amount RNA of independent preparation were pooled together to perform microarray analysis with an Affymetrix U133 chip. This dataset has been disposed to Gene Expression Omnibus (GEO) and assigned an accession number of GSE49184. There were 388 genes up-regulated and 343 genes down-regulated over 3-fold upon GAS2DN expression.

**Figure 7:**
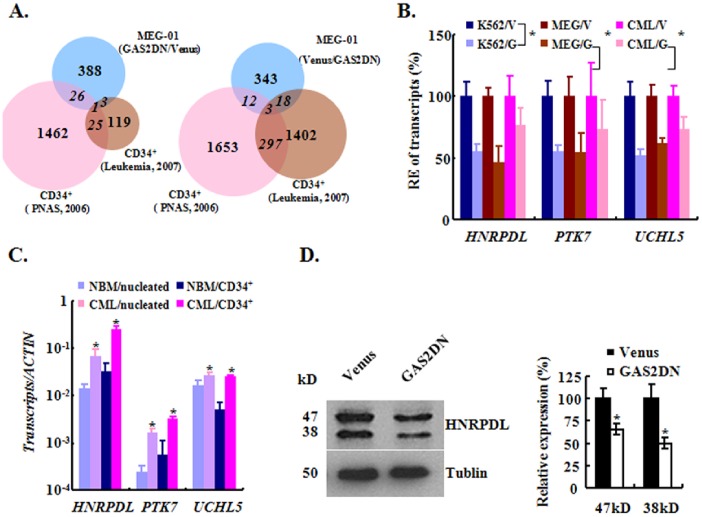
The transcriptome analysis of GAS2DN transduced cells. (A) Van-diagram of meta-analysis of the gene expression data. The differentially expressed genes identified in MEG-01 cells (blue) upon GAS2DN were compared with 2 public accessible datasets, namely the comparison between CML and NBM CD34^+^ cells (brown) and the comparison between CD34^+^ cells in CP and those in BC (purple). The 388 up-regulated genes in MEG-01 cells upon GAS2DN was compared with the 1462 genes down-regulated genes in CD34^+^ cells along disease progress and 119 genes down-regulated genes comparing CD34^+^ cells in CML versus NBM (left panel). Conversely, The 343 down-regulated genes in MEG-01 cells upon GAS2DN was compared with the 1653 genes up-regulated genes in CD34^+^ cells along disease progress and 1402 genes up-regulated genes comparing CD34^+^ cells in CML versus NBM (right panel). The numbers of genes for comparisons were shown in the middle of each pie, and the numbers of overrepresented genes were indicated in italic. (B) The relative gene expression of *HNRPDL, PTK7* and *UCHL5* in GAS2DN transduced K562, MEG-01 and primary CML cells (n = 3) was compared to that of control cells. (C) The gene expression of *HNRPDL, PTK7* and *UCHL5* in CML and normal bone marrow cells was assessed. For the nucleated cells, the samples of 7 individual healthy donor and 23 CML patients were used; for CD34^+^ cells, the samples of 4 individual healthy donors and 8 CML patients were used. (D) The protein expression of HNRPDL was analyzed with western blot. A representative graph was shown (left panel) and, the quantitative relative expression of both isoforms of HNRPDL was summarized from 3 independent replicates (right panel). RE, relative expression. *mean *p*<0.05, which was estimated with student *t*-test in a two-tailed fashion.

In order to narrow down the candidates for further validation and functional analysis, we performed meta-comparison of our data with 2 sets of public accessible array data (GSE5550 and GSE11889) [16], [17]. One set of data described the comparison between CD34^+^ cells from CML patients (n = 9) and those from NBM or mobilized peripheral blood (n = 8). 1402 genes were up-regulated in CML cells while 119 genes down-regulated significantly compared to normal control cells. The other set of data compared the genetic signature between CD34^+^ cells from CML patients in CP (n = 42) and those in BC (n = 32). 1653 genes was up-regulated in CML in BC while 1462 genes down-regulated compared to CML in CP. The up-regulated genes upon GAS2DN in our data were compared with the down-regulated genes in CML CD34^+^ cells compared to normal counterparts or the down-regulated genes in CD34^+^ cells along with the disease progress from CP to BC to identify the common genes; conversely the down-regulated genes upon GAS2DN were compared with the up-regulated genes in CML CD34^+^ cells versus NBM CD34^+^ cells or up-regulated genes along the disease progress to identify the overlapped ones. Together, we identified 55 common genes between our data and public database, which were listed in Table S3 in File S1 and illustrated in the Van-diagrams (Figure 7A).

We performed Q-RT-PCR and validated that *HNRPDL*, *UCHL5* and *PTK7* were consistently down-regulated upon GAS2DN in K562, MEG-01 and 3 individual primary CML cells (Figure 7B). Next we used both nucleated cells and CD34^+^ cells from CML patients in CP and healthy donors to assess the expression of these genes, and the results showed that *HNRPDL*, *UCHL5* and *PTK7* had significantly higher expression in CML cells than that in NBM cells (Figure 7C). Western blot showed that HNRPDL had 2 isoforms (47 and 38 kD) in K652 cells and both of them were reduced significantly when GAS2DN was expressed (Figure 7D).

### Knockdown of HNRPDL Inhibits the Growth of CML cells

We chose HNRPDL to perform functional study, which was firstly identified in K562 cells and belonged to heterogenous nuclear nucleoriboproteins (hnRNP) [18]. Many members of hnRNP, such as FUS, hnRNP A, hnRNP K, hnRNP E2 and La/SSB have been reported to contribute to the process of disease progression by either enhancing the proliferation or blocking the differentiation pathway of leukemic cells, through their ability to regulate the translation efficiency of target mRNAs [19]–[24]. Recently, it was reported that HNRPDL was significantly elevated in androgen-independent (AI) prostate cancer tissues compared to that in androgen-dependent (AD) prostate cancer. The forced expression of HNRPDL in LNCaP cells led to abnormal proliferation possibly due to the induction of EGF-R expression [25].

**Figure 8:**
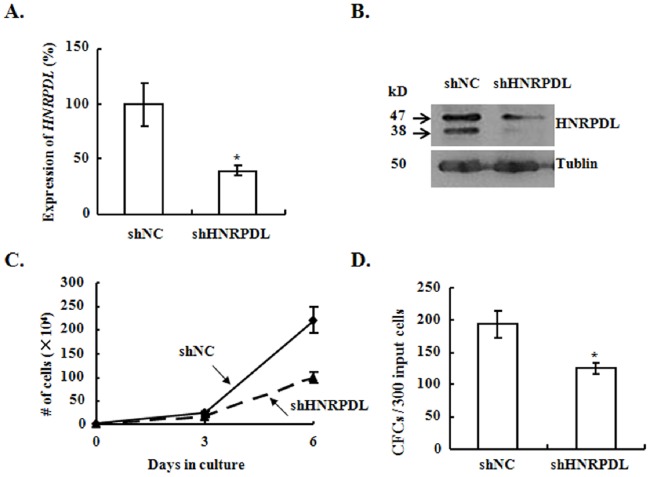
The knockdown of *HNRPDL* inhibits the growth of K562 cells. (A) The expression of *HNRPDL* was assessed with Q-RT-PCR in the virally transduced cells. (B) The expression of HNRPDL was analyzed with western blot. (C) & (D) The effect of HNRPDL silence on the proliferation and CFC production was analyzed. *mean *p*<0.05, which was estimated from 3 independent experiments with student *t*-test in a two-tailed fashion.

We utilized lentiviral vector to deliver shRNA against *HNRPDL* into K562 cells and found the loss of nearly 60% of the mRNA expression and the reduction of both isoforms of HNRPDL proteins (Figure 8A & 8B). The proliferation and CFC production of K562 cells was suppressed when HNRPDL was silenced (Figure 8C & 8D). These results demonstrated that knockdown of *HNRPDL* was sufficient to cause the growth inhibition of K562 cells, which suggested that GAS2DN inhibited the growth of CML cells through affecting the expression of multiple genes including *HNRPDL*.

## Discussion

The formation of *BCR-ABL* fusion gene is the molecular hallmark of CML, which encodes BCR-ABL tyrosine kinase with constitutive activity. The TKI can treat the disease in CP effectively, however unless the patients receive life-long TKI, the leukemia will recur eventually [1], [2]. Thus investigation of comprehensive molecular mechanisms of CML cells and the identification of novel therapeutic targets is still of importance for the management of the disease.

In this study, we clearly demonstrated higher transcript and protein expression of GAS2 in nucleated cells and the CD34^+^ cells from CML patients compared to that from healthy donors. To explore the functional role of GAS2 in CML cells, we suppressed GAS2 with both RNAi and the expression of GAS2DN. Interestingly, both approaches successfully inhibited the growth of CML cells, suggesting that GAS2 was required for the growth of these cells. Since the silence of *CALPAIN2* rescued the decreased cell proliferation caused by GAS2DN expression, we concluded that the inhibition of CML cells growth was partially calpain dependent. The suppressive function of GAS2DN was also applicable to the primary CD34^+^ CML progenitor cells; which was the first time to demonstrate that GAS2DN inhibited the growth of progenitor cells from CML patients. Importantly, we performed similar experiments with CD34^+^ cells from NBM, and GAS2DN did not inhibit the growth of normal cells as effectively as the leukemic cell. This relatively specific inhibition of CML cells and the enhanced IM sensitivity of K562 cells caused by GAS2 targeting made GAS2 an interesting therapeutic target for the disease. In addition, GAS2 was not only deregulated in CML but also in other myeloid proliferative diseases including idiopathic myelofibrosis, polycythemia vera and essential thrombocythemia [26], thus it was interesting to investigate whether GAS2 was required for the improper growth of cells from these hematological disorders as well.

Previous studies have suggested that targeting GAS2 against cancer cells is due to the degradation of beta-catenin and the reduction of its transcriptional activity [13], [14]. Indeed, beta-catenin is implicated in CML in many ways. The granulocyt-marcrophage progenitor cells in blast crisis or IM resistant CML had elevated nuclear localized beta-catenin, which was proved to be critical for the self-renewal of CML primitive cells *in vitro* [27]. BCR-ABL could directly interact and phosphorylate beta-catenin at Y86 and Y654 residues and the inhibition of beta-catenin with RNAi suppressed the growth of CML cells in synergism with IM [28]. Recently, the genetic and pharmacologic inhibition of beta-catenin was shown to effectively eradicate IM resistant leukemic stem cells in CML mice model [29]. However, we did not detect the reduction of beta-catenin upon RNAi or expression of GAS2DN. Indeed, we did not detect the transcriptional activity of beta-catenin in K562 and MEG-01 cells either. Thus we reasoned that there could be beta-catenin independent ways for GAS2DN to have its anti-cancer effect.

In the present study, we performed transcriptome analysis to obtain clues of how GAS2DN inhibited CML cells. Through the meta-comparison with array data generated by Diaz-Blanco E. *et al*. and Radich J. *et al*. [16], [17], we focused on a tiny set of overrepresented genes. The Q-RT-PCR data validated 3 candidates, namely *HNRPDL*, *PTK7* and *UCHL5*. Interestingly, all these 3 genes were up-regulated in both nucleated cells and CD34^+^ cells from CML patients compared to those from healthy donors, which suggested their potential oncogenic activities in CML cells. The knockdown experiment with K562 cells suggested that HNRPDL like many other hnRNPs possibly played an important role in the regulation of the growth of CML cells. Vigorous investigations about the suppressive effect of HNRPDL silence on primitive CML cells in parallel with that on the normal primitive hematopoietic cells would be valuable to fully address this point in the future.

PTK7 (protein tyrosine kinase 7) is an orphan tyrosine kinase receptor, first identified in colon carcinoma cells and also named colon carcinoma kinase-4 (CCK-4) [30]. PTK7 was identified as a versatile co-receptor for various signal pathways including Wnt, VEGF and Semaphorin, to regulate planar cell polarity, angiogenesis and cell migration [31]–[34]. PTK7 was found to be deregulated in cancer cells [35]–[38]. A recent report described a sequential cleavage of ADAM17 and gamma-secretase to generate the nucleus localized C-terminus PTK7, which could promote the cell proliferation, migration, and anchorage-independent colony formation [38]. PTK7 is expressed in some human acute myeloid leukemia (AML) patients, who are more resistant to anthracycline-based frontline therapy with a significantly reduced leukemia-free survival. PTK7 in AML cells promotes their migration, survival, and resistance to anthracycline-induced apoptosis. Conversely, primary AML blasts are sensitized to anthracycline-mediated cell death by a recombinant soluble PTK7-Fc protein [36]. Based on the fact that PTK7 plays an important role in AML, it is interesting to investigate how it modulates the property of CML cells and whether it’s implicated in the GAS2 targeting induced CML suppression.

UCHL5 belongs to proteasome deubiquitinase (DUB). The ubiquitin-proteasome system is a conserved pathway regulating multiple biological processes including protein turnover, DNA repair, and intracellular trafficking. The 26S proteasome particle contains two subunits, the 20S core particle (CP) and 19S regulatory particle (RP). The elevated activity of proteasome in CML was reported previously, and the inhibitors targeting 20S proteasome such as bortezomib achieved growth inhibition in synergism with IM [39], [40]. Recently several DUBs have been shown to be involved in cancer progression [41], for example UCHL5 is up-regulated in human cancer including cervical carcinoma and esophageal squamous cell carcinoma (ESCC), and its expression has been identified as a poor prognostic indicator for ESCC as well [42], [43]. UCHL5 has been shown to have the anti-apoptosis effect in A549 cells [44]. The inhibitors targeting DUBs as novel anti-cancer therapies have been developed [45]–[47], such as both WP1130 and b-AP15 against UCHL5. As we have shown the up-regulation of UCHL5 in CML cells, it is interesting to assess the efficacy of these DUB inhibitors to suppress CML cells.

Overall, we have demonstrated the up-regulation of GAS2 in CML and it is required for the growth of CML cells. Targeting GAS2 causes relatively specific inhibition of CML cells and enhances their IM sensitivity, which suggests GAS2 is a novel therapeutic target as well. Our data also suggest a beta-catenin independent way for GAS2DN to inhibit the CML cell growth through affecting the expression of a subset of genes including *HNRPDL*. Further investigation of the function and the underlying mechanism of GAS2 in CML would benefit the management of the disease, and shed light on the multiple facets of the biological function of GAS2.

## Supporting Information

File S1 **Supporting information file containing Figures S1–S4 and Tables S1–S3.** **Figure S1.** GAS2 rescues the reduced CFC production upon GAS2 silence. K562 cells transduced with various lentiviral vectors were purified with cell sorter, and then their CFC production were compared. The data was presented as mean ± SEM with 3 independent experiments. *mean *p*<0.05, which was estimated with student *t*-test in a two-tailed fashion. **Figure S2.** The measurement of calpain activity upon CALPAIN2 silence in K562 cells. The relative calpain activities of control and CALPAIN2 silenced cells were compared. The data was presented as mean ± SEM with 3 independent experiments. *mean *p*<0.05, which was estimated with student *t*-test in a two-tailed fashion. **Figure S3.** The effect of calpain inhibitor on the proliferation of GAS2DN expressed K562 cells. The proliferation of Venus and GAS2DN transduced cells with and without PD150606 (20 µM, the calpain inhibitor) was measured. The data was presented as mean ± SEM with 3 independent experiments. *mean *p*<0.05, which was estimated with student *t*-test in a two-tailed fashion. **Figure S4.** CD34 expression of the transduced normal and leukemic cells. The FACS profiles showed CD34 expression of various transduced normal bone marrow (NBM) and chronic myeloid leukemia (CML) cells. The number indicated the average percentage of CD34^+^YFP^+^ cells from 2 (normal cells) and 3 (leukemic cells) individual samples, respectively. These double positive cells were sorted for colony-forming cell (CFC) assay. **Table S1.** The clinical characteristics of chronic myeloid leukemia patients in this study. **Table S2.** Primers used in this study. **Table S3.** The overrepresented genes found in previously published data. The description of the overrepresented genes identified between the dataset generated in this study (GSE49184) and the published datasets (GSE5550 and GSE11889).
(DOC)
