# Supplementary material for: Growth Arrest Specific 2 Is Up-Regulated in Chronic Myeloid Leukemia Cells and Required for Their Growth
Source: PLoS One. 2014 Jan 21;9(1):e86195. doi: 10.1371/journal.pone.0086195 (PMC3897655; doi:10.1371/journal.pone.0086195)
Supplement: File S1 — Supporting information file containing Figures S1–S4 and Tables S1–S3. Figure S1. GAS2 rescues the reduced CFC production upon GAS2 silence. K562 cells transduced with various lentiviral vectors were purified with cell sorter, and then their CFC production were compared. The data was presented as mean ± SEM with 3 independent experiments. *mean p<0.05, which was estimated with student t-test in a two-tailed fashion. Figure S2. The measurement of calpain activity upon CALPAIN2 silence in K562 cells. The relative calpain activities of control and CALPAIN2 silenced cells were compared. The data was presented as mean ± SEM with 3 independent experiments. *mean p<0.05, which was estimated with student t-test in a two-tailed fashion. Figure S3. The effect of calpain inhibitor on the proliferation of GAS2DN expressed K562 cells. The proliferation of Venus and GAS2DN transduced cells with and without PD150606 (20 µM, the calpain inhibitor) was measured. The data was presented as mean ± SEM with 3 independent experiments. *mean p<0.05, which was estimated with student t-test in a two-tailed fashion. Figure S4. CD34 expression of the transduced normal and leukemic cells. The FACS profiles showed CD34 expression of various transduced normal bone marrow (NBM) and chronic myeloid leukemia (CML) cells. The number indicated the average percentage of CD34+YFP+ cells from 2 (normal cells) and 3 (leukemic cells) individual samples, respectively. These double positive cells were sorted for colony-forming cell (CFC) assay. Table S1. The clinical characteristics of chronic myeloid leukemia patients in this study. Table S2. Primers used in this study. Table S3. The overrepresented genes found in previously published data. The description of the overrepresented genes identified between the dataset generated in this study (GSE49184) and the published datasets (GSE5550 and GSE11889). (DOC) [file pone.0086195.s001.doc]

**Supporting Information**


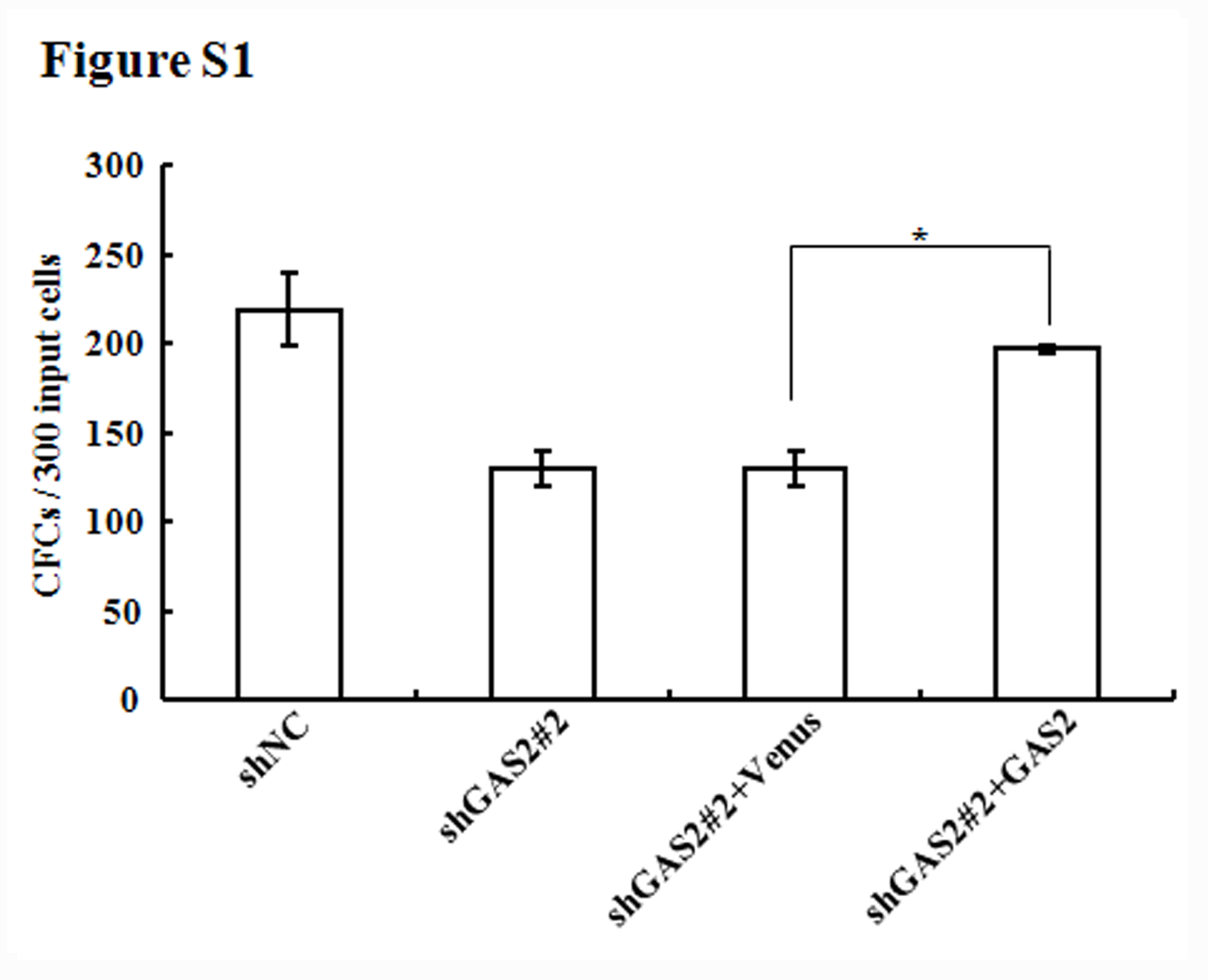


**Figure S1. GAS2 rescues the reduced CFC production upon GAS2 silence.** K562 cells transduced with various lentiviral vectors were purified with cell sorter, and then their CFC production were compared. The data was presented as mean ± SEM with 3 independent experiments. * mean *p* < 0.05, which was estimated with student *t*-test in a two-tailed fashion.

**
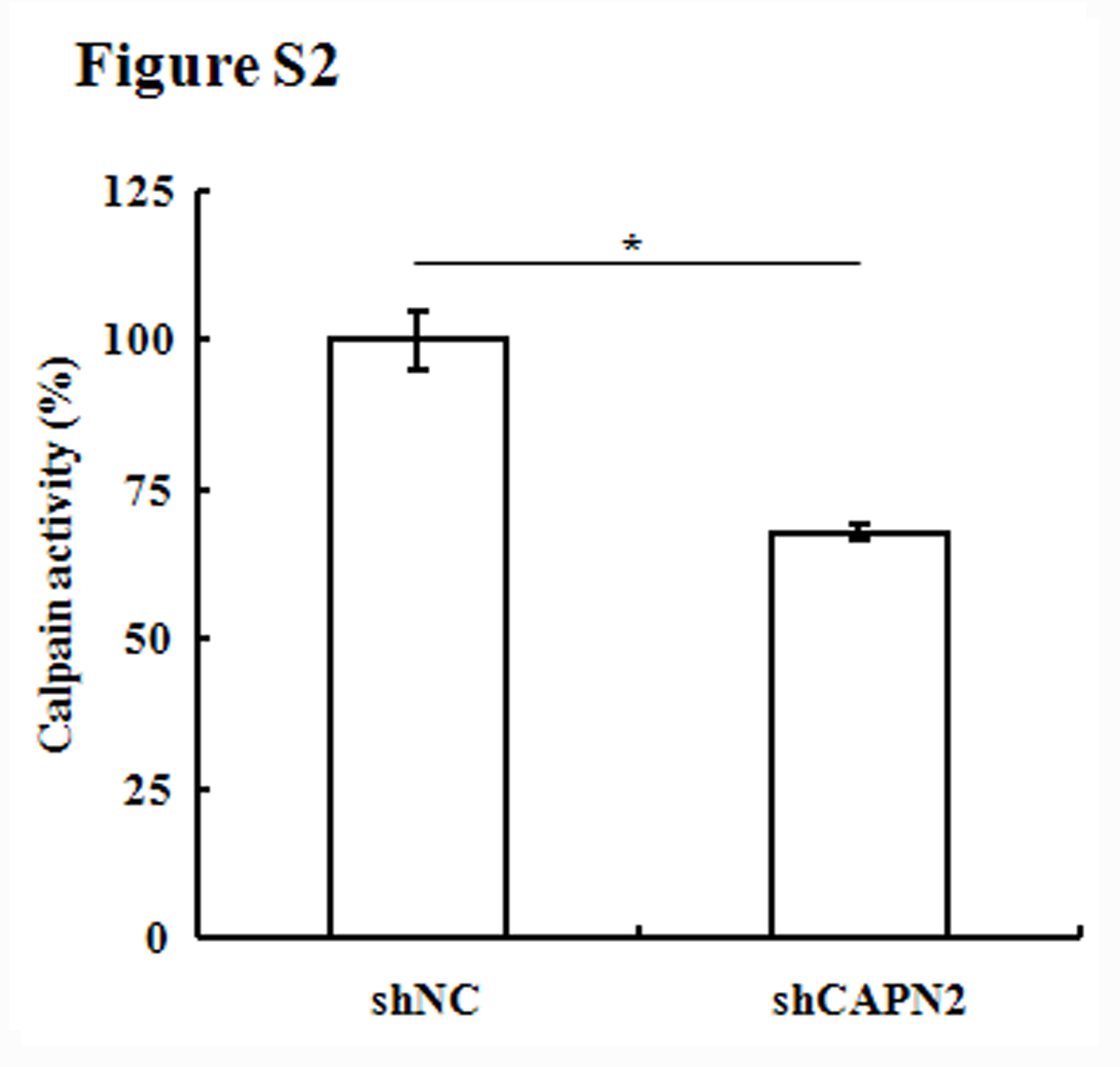
**

**Figure S2. The measurement of calpain activity upon CALPAIN2 silence in K562 cells.** The relative calpain activities of control and CALPAIN2 silenced cells were compared. The data was presented as mean ± SEM with 3 independent experiments. * mean *p* < 0.05, which was estimated with student *t*-test in a two-tailed fashion.

**
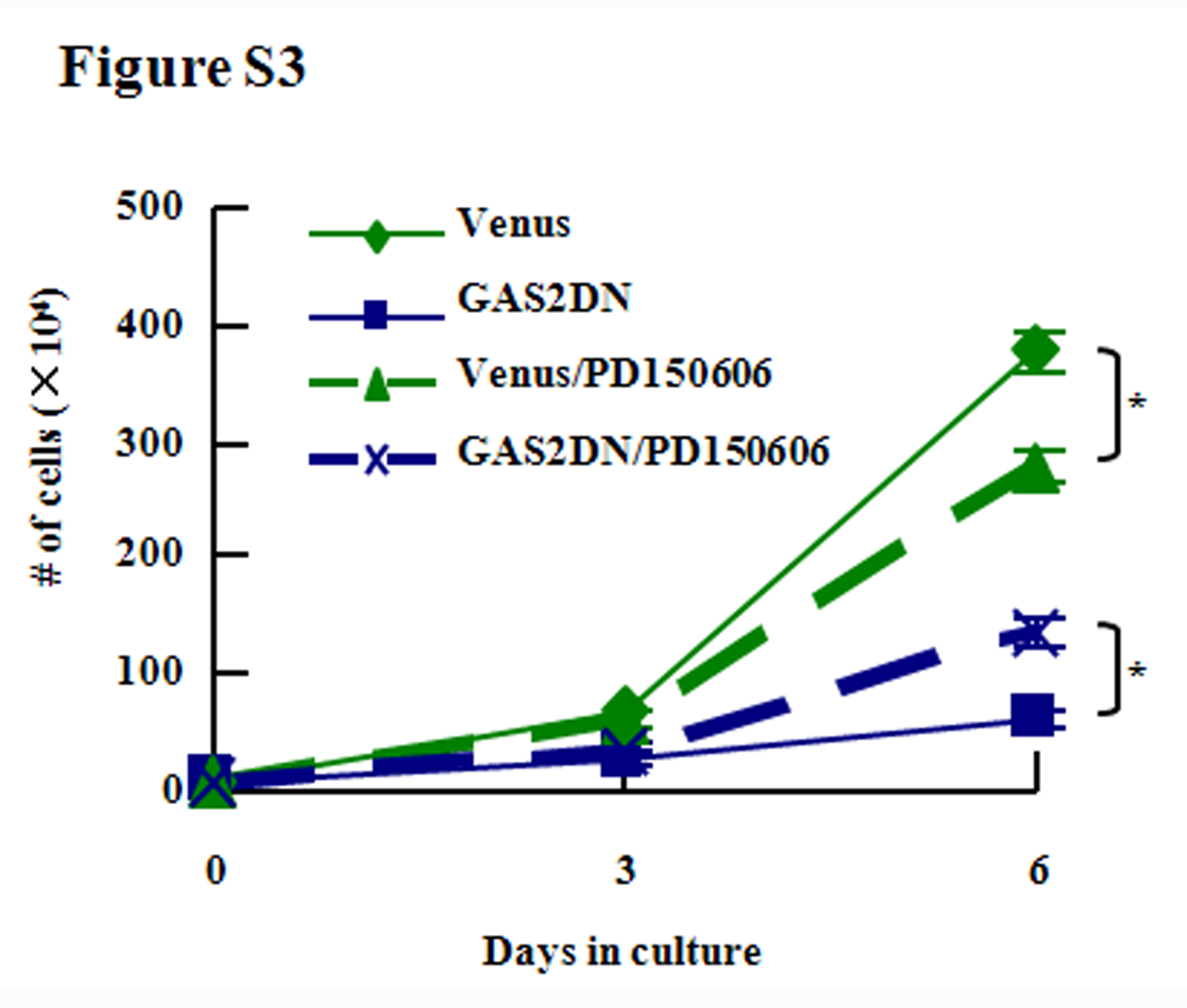
**

**Figure S3. The effect of calpain inhibitor on the proliferation of GAS2DN expressed K562 cells.** The proliferation of Venus and GAS2DN transduced cells with and without PD150606 (20 μM, the calpain inhibitor) was measured. The data was presented as mean ± SEM with 3 independent experiments. * mean *p* < 0.05, which was estimated with student *t*-test in a two-tailed fashion.


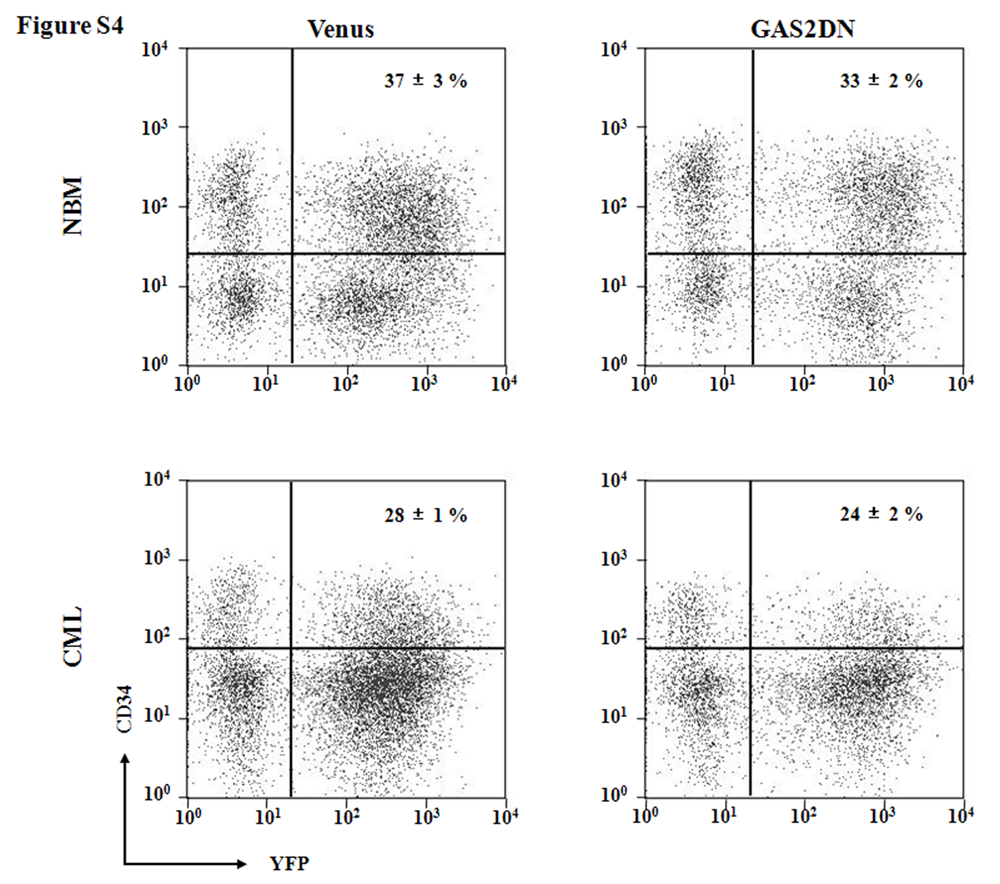


**Figure S4. CD34 expression of the transduced normal and leukemic cells.** The FACS profiles showed CD34 expression of various transduced normal bone marrow (NBM) and chronic myeloid leukemia (CML) cells. The number indicated the average percentage of CD34^+^YFP^+^ cells from 2 (normal cells) and 3 (leukemic cells) individual samples, respectively. These double positive cells were sorted for colony-forming cell (CFC) assay.

**Table S1. The clinical characteristics of chronic myeloid leukemia patients in this study.**

| **Sex** | **Age (years)** | **WBC (×10^9^/L)** | **Hb (g/L)** | **PLT (×10^9^/L)** |
| --- | --- | --- | --- | --- |
| Male, 21  Female, 12 | 42.5  (10-65) | 172.5  (41.5-463.3) | 98.4  (72-168) | 449  (87-899) |

*a*, The median of each characteristic was presented and the number in bracket indicated the range of the data.

**Table S2. Primers used in this study.**

| **Gene symbol** | **Primers** | | **Amplicon**  **(bp)** |
| --- | --- | --- | --- |
| *ACTIN* | F | CACCATTGGCAATGAGCGGTTCC | 90 |
|  | R | GTAGTTTCGTGGATGCCACAGG |  |
| *GAS2* | F | GCAACCCAGAGAAGTGTGTCT | 74 |
|  | R | CAGGAGGCTCCACACCAT |  |
| *HNRPDL* | F | AGAATCAGCAGGATGACGGTA | 95 |
|  | R | ACTTCCCCAAATCGAGACAAGT |  |
| *PTK7* | F | GGTCTAGCCTGCAGCCCATC | 102 |
|  | R | TACCAGGGTCTCTGCCACTC |  |
| *UCHL5* | F | CAAGTGGCAGCCAGGAGAAGA | 114 |
|  | R | TCACTATGGCTTGAGTAGCACA |  |
| *CALPAIN2* | F | GCAGCCATTGCCTCCCTCAC | 131 |
|  | R | ACCTCCACCCACTCGCCGTA |  |

**Table S3. The overrepresented genes found in previously published data.**

| **Gene Symbol** | **Gene ID** | **Fold-change (GAS2SN vs. Venus)** | **CD34^+^ cells (CML vs. NBM)*^b^*** | **CD34^+^ cells (BC vs. CP)*^c^*** |
| --- | --- | --- | --- | --- |
| *FGF14* | 2259 | 66.5014 |  | + |
| *CDC14B* | 8555 | 48.0236 |  | + |
| *IGL* | 3535 | 42.8378 |  | + |
| *TBCD* | 6904 | 40.4666 |  | + |
| *IGSF6* | 10261 | 30.9737 |  | + |
| *PDE4DIP* | 9659 | 29.4273 |  | + |
| *GSN* | 2934 | 24.1921 |  | + |
| *ARPC5* | 10092 | 16.2994 |  | + |
| *LILRA2* | 11027 | 14.6811 |  | + |
| *ITPK1* | 3705 | 10.9878 |  | + |
| *ALOX5* | 240 | 10.2312 |  | + |
| *NDST2* | 8509 | 9.7392 |  | + |
| *GM2A* | 2760 | 7.7664 |  | + |
| *ZNF398* | 57541 | 7.7206 |  | + |
| *MYL6* | 4637 | 6.8788 |  | + |
| *OPRL1* | 4987 | 6.5454 |  | + |
| *ALDH3B1* | 221 | 5.4456 |  | + |
| *PTGES* | 9536 | 5.4422 |  | + |
| *EEF1A1* | 1915 | 5.192 | + |  |
| *CSH1* | 1442 | 4.2725 |  | + |
| *ALPP* | 250 | 4.8704 |  | + |
| *PSEN1* | 5663 | 4.7817 |  | + |
| *PSG6* | 5675 | 4.7684 |  | + |
| *CSTA* | 1475 | 3.8397 | + | + |
| *TERT* | 7015 | 3.4389 | + |  |
| *PPAP2B* | 8613 | 3.2909 |  | + |
| *CEACAM1* | 634 | 3.1698 |  | + |
| *LIPE* | 3991 | 3.0691 |  | + |
| *NCBP1* | 4686 | 0.3259 | + |  |
| *TRIM16* | 10626 | 0.3256 | + |  |
| *PTPN13* | 5783 | 0.3201 | + |  |
| *PMS1* | 5378 | 0.319 | + |  |
| *HNRPDL* | 9987 | 0.3147 |  | + |
| *PTK7* | 5754 | 0.3116 |  | + |
| *MRS2L* | 57380 | 0.2961 |  | + |
| *QPCT* | 25797 | 0.2949 | + |  |
| *VAPA* | 9218 | 0.2749 | + |  |
| *SCARB1* | 949 | 0.2718 |  | + |
| *HSPA9B* | 3313 | 0.2704 | + |  |
| *FADS3* | 3995 | 0.2597 |  | + |
| *NMT2* | 9397 | 0.2594 | + | + |
| *BCL2L1* | 598 | 0.2523 | + |  |
| *SFRS12* | 140890 | 0.251 |  | + |
| *TAX1BP1* | 8887 | 0.2262 | + |  |
| *FDX1* | 2230 | 0.2018 |  | + |
| *SFRS2IP* | 9169 | 0.1947 | + |  |
| *GCLM* | 2730 | 0.1898 | + |  |
| *TRUB1* | 142940 | 0.1801 |  | + |
| *TTF2* | 8458 | 0.1704 | + |  |
| *TMOD3* | 29766 | 0.1662 | + |  |
| *NNT* | 23530 | 0.1635 | + | + |
| *FEZ2* | 9637 | 0.1417 | + |  |
| *MTHFD1* | 4522 | 0.1239 | + |  |
| *UCHL5* | 51377 | 0.1172 | + | + |
| *MTA3* | 57504 | 0.0395 |  | + |

*a,* “+” signified that the gene was overrepresented.

*b,* CD34^+^ cells from the Table S2 of Diaz-Blanco E *et al*. (Ref 17, GSE5550)

*c,* CD34^+^ cells from the Table S4 of Radich J *et al*. (Ref 16, GSE11889). BC, blast crisis; CP, chronic phase.
